# Value of Blood Count–Derived Inflammatory Markers for Evaluating Psoriasis Severity: Pilot Cross-Sectional Observational Study

**DOI:** 10.2196/86454

**Published:** 2026-05-14

**Authors:** Luyuan Wang, Xiaorui Zhang, Chuangang Xia, Wenyu Li, Qiuju Li, Wen Xiao, Youkun Lin

**Affiliations:** 1 Department of Dermatology and Venereology, Fourth Affiliated Hospital of Guangxi Medical University, Liuzhou, Guangxi, China; 2 First Clinical Medical College, Guangxi Medical University, Nanning, Guangxi, China; 3 Department of Dermatology and Venereology, First Affiliated Hospital of Guangxi Medical University, 6 Shuangyong Road, Nanning, Guangxi, 530021, China, 86 0771-5356514

**Keywords:** psoriasis, systemic inflammation, interleukins, Psoriasis Area and Severity Index score, PASI score, disease assessment

## Abstract

**Background:**

Objective indicators are urgently needed to evaluate and monitor disease progression in patients with psoriasis.

**Objective:**

This study aimed to verify the correlations between blood count–derived inflammatory markers and the Psoriasis Area and Severity Index (PASI) among patients with psoriasis and explore the value of applying the PASI in combination with proinflammatory factors.

**Methods:**

This was a cross-sectional observational study that enrolled 719 patients from 2 tertiary hospitals. Receiver operating characteristic curve analysis and binary logistic regression models were applied to assess the evaluative power of blood count–derived inflammatory markers and their consistency with the PASI for stratifying psoriasis severity. The association with the PASI and the combination with proinflammatory factors of the blood count–derived inflammatory markers in 60 patients were analyzed. The exploratory association between blood count–derived markers and proinflammatory factors was analyzed using product terms. To ensure robustness, multivariable combined models were evaluated using receiver operating characteristic curves and decision curve analysis. Model performance was further validated via calibration plots and a predictive nomogram, with the decision curve analysis net benefit axis increased to 1.0 for comprehensive visualization.

**Results:**

The area under the curve showed that the systemic immune-inflammation index (SII), systemic inflammation response index (SIRI), and aggregate index of systemic inflammation (AISI) were effective in reflecting psoriasis severity and showed advantages in patients with psoriasis complicated by arthritis and cardiovascular metabolic diseases. The comprehensive test showed quite appropriate consistency of the SIRI and PASI in distinguishing severity. The SII, SIRI, and AISI were significantly correlated with interleukin (IL)-6 in lesions (all *P*<.05), and the combinations of these indices with IL-6, IL-1, and IL-17 were also significantly correlated with the PASI (all *P*<.05).

**Conclusions:**

Blood count–derived inflammatory markers could better reflect the inflammation of patients with psoriasis. The SII, SIRI, and AISI have important clinical significance in evaluating disease severity. The combination with proinflammatory factors showed an advantage.

## Introduction

### Background of Psoriasis and Its Clinical Challenges

Psoriasis is a common chronic inflammatory skin disease that is associated with psoriatic arthritis (PsA), obesity, dyslipidemia, hypertension, type 2 diabetes, and cardiovascular disease [1]. Blood cells interact with proinflammatory factors to regulate the progression of psoriasis. The increase in and prolongation of the systemic inflammatory state in patients may be important determinants of the severity, progression, comorbidities, and outcome of psoriasis. Presently, the treatment model for psoriasis is reactive; therefore, a more proactive and risk-stratified management approach is needed. Early intervention and effective treatment can alleviate the severity of psoriasis by reducing inflammation levels and may also reduce or prevent diseases such as cardiometabolic diseases, as well as irreversible damage and disability. There is increasing acknowledgment that a single variable is rarely likely to be a good predictor of disease progression [2]; therefore, new parameters are required due to the lack of objective evaluation criteria, insufficient evaluation of chronic inflammation–causing disease, and differences between clinicians.

### Blood Count–Derived Inflammatory Markers: Research Status

Complete blood count parameters have gained recognition as valuable biomarkers for a range of inflammatory conditions, primarily because of their widespread availability and cost-effectiveness. In one study, compared with the healthy control group, patients with psoriasis had higher total white blood cell, neutrophil, and platelet count; neutrophil-to-lymphocyte ratio (NLR); and platelet-to-lymphocyte ratio (PLR) but lower lymphocyte counts. However, there was no significant correlation between the Psoriasis Area and Severity Index (PASI) score and either the NLR or PLR [3]. In another study, the systemic immune-inflammation index (SII) and systemic inflammation response index (SIRI) were powerful prognostic indicators of poor outcomes in patients with cancer and were promising tools for treatment strategy decisions [4,5]. The SIRI and aggregate index of systemic inflammation (AISI) can be useful for the early identification of patients at risk of prolonged hospital stay in open elective thoracic surgery [6]. In another study, the NLR, PLR, monocyte-to-lymphocyte ratio (MLR), SII, SIRI, and AISI were referred to as the blood count–derived inflammatory markers [7]. Both the SII and SIRI have been shown to statistically significantly decrease in patients with psoriasis after only 3 months of treatment with biologics [8,9], and a low correlation between the reduction in PASI scores and PLR, SII, and SIRI values has been observed [9]. The AISI was first reported to be correlated with liver fibrosis severity in psoriasis vulgaris [10]. The AISI, SII, and SIRI have been positively correlated with disease severity in psoriasis [7].

### Research Gaps and Study Rationale

Overall, there are limited studies on the correlation between blood count–derived inflammatory markers and psoriasis. Some studies have had small sample sizes, and there are limited studies that have stratified patients with psoriasis by severity based on PASI score and investigated blood count–derived inflammatory markers. Conventional severity assessment tools, including PASI and body surface area, are insufficient to reflect the underlying pathophysiological features and comprehensive disease course of psoriasis [11]; moreover, the combination of blood count–derived inflammatory markers of psoriasis and proinflammatory factors remains under-studied.

## Methods

### Patients and Data Sources

This study enrolled patients with psoriasis between June 2, 2021, and February 2, 2024, from the Department of Dermatology and Venereology of the First Affiliated Hospital and Fourth Affiliated Hospital of Guangxi Medical University. All patients were diagnosed based on clinical or histopathological manifestations of chronic plaque-type psoriasis. Data on baseline demographics; clinical characteristics; concomitant joint arthritis; and scalp, genital, lower leg, and nail involvement were recorded in the clinic. The disease duration was defined as the period from the first diagnosis of psoriasis by a dermatologist until the present. The inclusion criteria for patients were as follows: (1) patients with chronic plaque-type psoriasis and (2) age of more than 18 years. The PASI was assessed by 3 clinical physicians who had undergone unified training. Exclusion criteria included pregnancy or breastfeeding, acute systemic infection, immunodeficiency, malignant tumors, hematologic disorders, and clinically significant anemia (eg, iron deficiency anemia). Additionally, patients receiving systemic treatments within 1 month preceding enrollment were excluded to ensure a stable baseline and complete blood counts. Standard blood tests were performed for all patients before initiation of treatment, and the results were obtained from the Department of Clinical Laboratory, First Affiliated Hospital and Fourth Affiliated Hospital of Guangxi Medical University. The First Affiliated Hospital cohort served as the derivation set, whereas the Fourth Affiliated Hospital cohort functioned as the independent validation set.

### Ethical Considerations

This study was approved by the ethics committee of the Fourth Affiliated Hospital of Guangxi Medical University (approval KY2024508) and conducted according to the principles of the Declaration of Helsinki. Written informed consent was obtained from all participants.

### Definition of Blood Count–Derived Inflammatory Markers

The NLR was calculated as absolute neutrophil count (× 10^9^/L)/absolute lymphocyte count (× 10^9^/L), the PLR was calculated as absolute platelet count (× 10^9^/L)/absolute lymphocyte count (× 10^9^/L), the MLR was calculated as absolute monocyte count (× 10^9^/L)/absolute lymphocyte count (× 10^9^/L), the SII was calculated as absolute neutrophil count (× 10^9^/L) × absolute platelet count (× 10^9^/L)/absolute lymphocyte count (× 10^9^/L), the SIRI was calculated as absolute neutrophil count (× 10^9^/L) × absolute monocyte count (× 10^9^/L)/absolute lymphocyte count (× 10^9^/L), and the AISI was calculated as absolute neutrophil count (× 10^9^/L) × absolute platelet count (× 10^9^/L) × absolute monocyte count (× 10^9^/L)/absolute lymphocyte count (× 10^9^/L).

### Materials

An antibody against interleukin (IL)-17A (catalog number GB11110-1-50; 1:300) was purchased from ServiceBio, an antibody against IL-1β (cat. no. GB11113-50; 1:300) was purchased from ServiceBio, an antibody against IL-6 (cat. no. YT5348; 1:300) was purchased from Solarbio, and a goat antirabbit secondary antibody (cat. no. 23303-100; 1:200) was purchased from ServiceBio.

### Histomorphometric Analysis and Immunohistochemical Staining

The skin lesions were biopsied at the Department of Dermatology and Venereology of the First Affiliated Hospital of Guangxi Medical University. The lesions were immediately fixed in 4% paraformaldehyde. The samples were embedded in paraffin, and 4 µm thick sections were obtained. Immunohistochemical staining was performed to detect the level of IL-17, IL-1β, and IL-6 from a consecutive sample of 60 patients with psoriasis using the respective antibodies (1:300 dilution) according to the manufacturer’s protocol. The histochemistry score (H-score) was adopted for the standardized evaluation of the histological expression of immunohistochemical markers. The specific calculation method was as follows: H-score (∑ (*pi* × *i*) = (percentage of weakly positive cells × 1) + (percentage of moderately positive cells × 2) + (percentage of strongly positive cells × 3), where *i* represents the classification of positive cell grades (strongly positive brownish cells count for 3 points). Positive cells were counted using the ImageJ software. Assessors were blinded to PASI score and blood count–derived inflammatory markers during H-score evaluation.

### Statistical Analysis

Statistical analysis was performed using SPSS (version 25.0; IBM Corp). Statistical descriptions of normal or approximately normal measures were expressed as means and SDs; significantly skewed measures were expressed as medians and IQRs. Furthermore, categorical variables were presented as absolute counts and percentages. Categorical variables were compared using the chi-square test. The optimal cutoff values were determined using the Youden index within the derivation cohort only. Agreement between marker-based and PASI-based classifications in the validation cohort was further assessed using the Cohen κ (with 95% CI) and Pearson chi-square test. The Youden index was calculated as sensitivity – (1 – specificity). The differences between the groups were evaluated using the Mann-Whitney *U* test. Kolmogorov-Smirnov and Shapiro-Wilk tests were used for inspecting the normal distribution. Binary logistic regression analysis was used to further test the results of the Mann-Whitney *U* test. Binary logistic regression included adjustments for BMI, smoking status, and comorbidities (Multimedia Appendix 1). To verify the robustness of these conclusions, a sensitivity analysis was conducted by re-evaluating the inflammatory markers exclusively in a subgroup of patients without any comorbidities (n=571). Due to the skewed distribution and disparate unit scales of the inflammatory markers, natural log-transformation (ln) was applied to improve model stability and interpretability. Extended multivariable models additionally adjusted for study center, disease duration, and PsA. To address potential institutional bias, a stratified receiver operating characteristic (ROC) curve analysis was performed for each hospital cohort, and study center was included as an additional covariate in the sensitivity analysis of the regression model. Comparisons of area under the curve (AUC) values between markers were performed using the DeLong test, with the Benjamini-Hochberg procedure applied to control the false discovery rate (FDR) for multiple comparisons. The Hosmer-Lemeshow test was used to evaluate the goodness of fit of the logistic regression model. Correlations were evaluated using the Spearman correlation coefficient. A *P* value of less than .05 was considered statistically significant. Granular subgroup and correlation analyses were designated as exploratory in nature. A PASI score of 10 or higher vs a PASI score of less than 10 was used as the reference standard for ROC and logistic regression analysis.

## Results

### Baseline Clinical and Demographic Characteristics

The clinical and demographic characteristics of all participants included in the study are shown in Table 1.

Overall, 545 patients were recruited from the Department of Dermatology and Venereology of the First Affiliated Hospital of Guangxi Medical University, and another 174 eligible patients were enrolled from the Department of Dermatology and Venereology of the Fourth Affiliated Hospital of Guangxi Medical University. Among the 719 patients, the lesions involved the scalp in 427 (59.4%) patients, arthritis in 59 (8.2%) patients, the nails in 171 (23.8%) patients, the genital area in 83 (11.5%) patients, and the lower leg in 106 (14.7%) patients. Overall, 148 participants had chronic systemic comorbidities, of whom 97 (65.5%) had cardiometabolic disorders.

**Table 1.** Characteristics of the patients with psoriasis.

Characteristic		Values
First Affiliated Hospital (n=545)		
Gender (woman), n (%)		141 (25.9)
Age (y), mean (SD)		43.14 (15.76)
BMI (kg/m^2^), median (IQR)		21.95 (20-23.74)
Ever smokers, n (%)		193 (35.4)
Psoriasis duration (y), median (IQR)		2 (1-6)
Specific body part involved, n (%)		
Scalp		304 (55.8)
Joint		48 (8.8)
Nails		121 (22.2)
Genitals		40 (7.3)
Lower leg		79 (14.5)
Comorbidity, n (%)		124 (22.8)
PASI^a^, median (IQR)		9 (5.4-16)
Fourth Affiliated Hospital (n=174)		
Gender (woman), n (%)		54 (31.0)
Age (y), mean (SD)		49.66 (14.85)
BMI (kg/m^2^), median (IQR)		24.22 (22.39-27.43)
Ever smokers, n (%)		36 (20.7)
Psoriasis duration (y), median (IQR)		3 (0.66-9)
Specific body part involved, n (%)		
Scalp		123 (70.7)
Joint		11 (6.3)
Nails		50 (28.7)
Genitals		43 (24.7)
Lower leg		27 (15.5)
Comorbidity, n (%)		24 (13.8)
Treatment, n (%)		
Secukinumab		100 (57.5)
Guselkumab		15 (8.6)
Ustekinumab		9 (5.2)
Methotrexate		31 (17.8)
Acitretin		32 (18.4)
Cyclosporin		22 (12.6)
Irregular treatment with secukinumab		9 (5.2)
PASI, median (IQR)		12.2 (6-21.73)

a PASI: Psoriasis Area and Severity Index.

### Blood Count–Derived Inflammatory Markers and Psoriasis Severity

**Figure 1.**
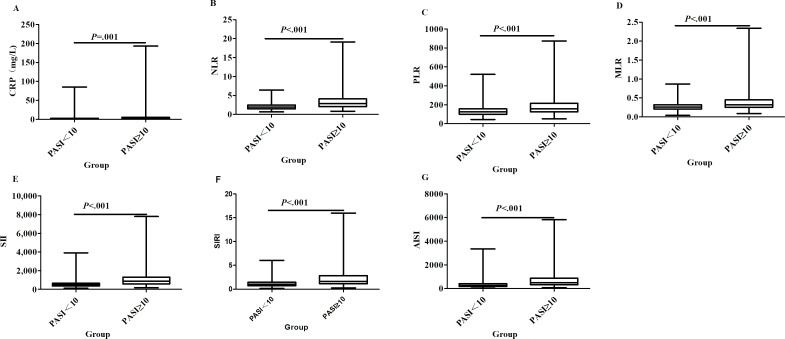
The differential expression of the blood count–derived indexes in patients with moderate and severe psoriasis. AISI: aggregate index of systemic inflammation; CRP: C-reactive protein; MLR: monocyte-to-lymphocyte ratio; NLR: neutrophil-to-lymphocyte ratio; PASI: Psoriasis Area and Severity Index; PLR: platelet-to-lymphocyte ratio; SII: systemic immune-inflammation index; SIRI: systemic inflammation response index.

**Figure 2.**
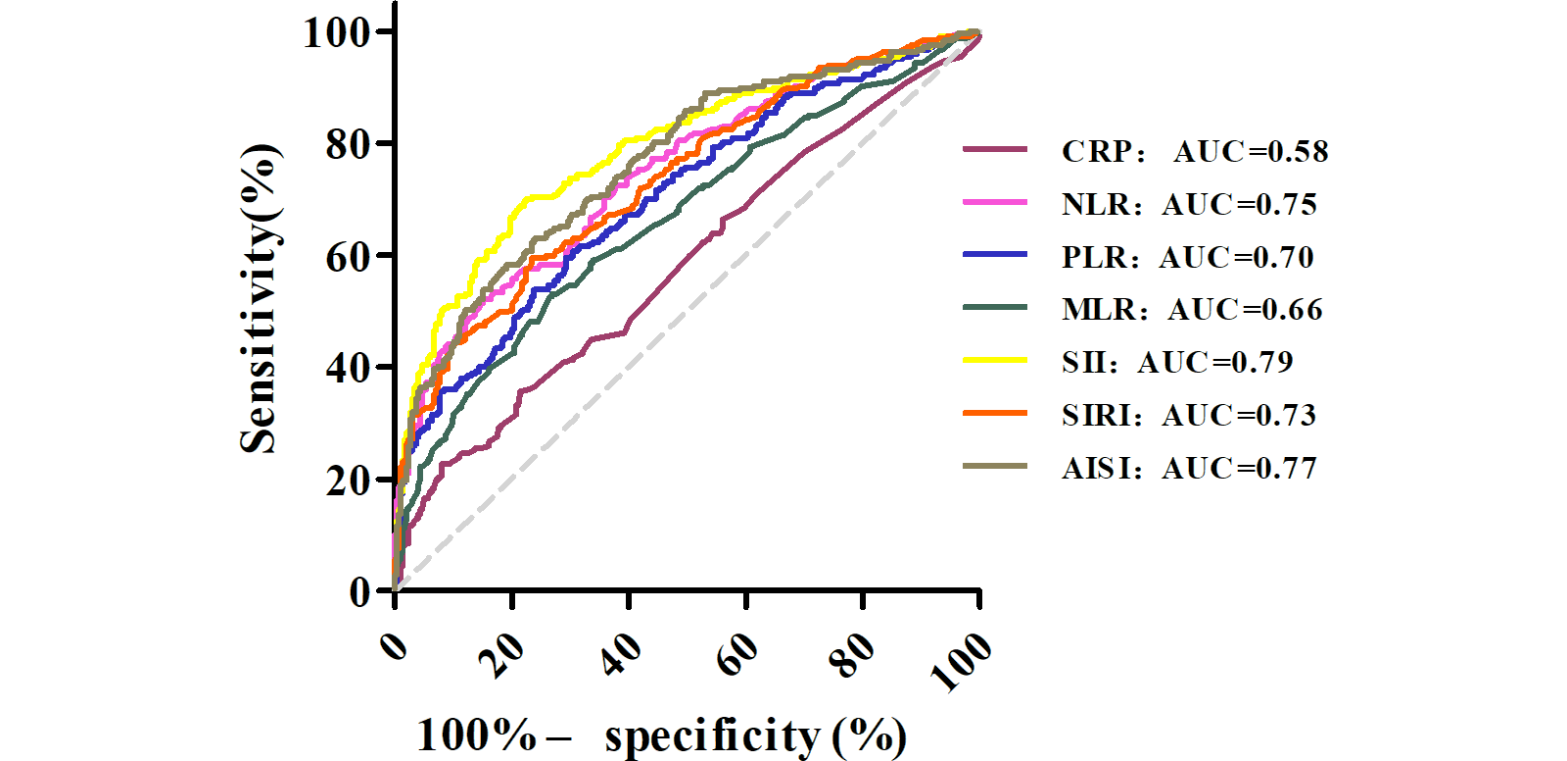
Receiver operating characteristic curve analysis for assessing the performance of the blood count–derived indexes in the prediction of psoriasis activation. AISI: aggregate index of systemic inflammation; AUC: area under the curve; CRP: C-reactive protein; MLR: monocyte-to-lymphocyte ratio; NLR: neutrophil-to-lymphocyte ratio; PLR: platelet-to-lymphocyte ratio; SII: systemic immune-inflammation index; SIRI: systemic inflammation response index.

Of the 545 patients with psoriasis in the First Affiliated Hospital, 298 (54.7%) were in the group with a PASI score of less than 10, and 247 (45.3%) were in the group with a PASI score of 10 or higher. NLR, PLR, MLR, SII, SIRI, and AISI were all significantly higher in the group with a PASI score of 10 or higher than in the group with a PASI score of less than 10 (*P*<.001 in all cases; Figure 1). The higher the blood count–derived inflammatory markers, the higher the psoriasis severity. The results of the ROC curve analyses evaluating the ability of blood count–derived indexes (NLR, PLR, MLR, SII, SIRI, and AISI) to be associated with psoriasis disease severity are presented in Figure 2. The AUC values were 0.58 (95% CI 0.53-0.63) for C-reactive protein (CRP), 0.75 (95% CI 0.70-0.79) for NLR, 0.70 (95% CI 0.66-0.75) for PLR, 0.66 (95% CI 0.62-0.71) for MLR, 0.79 (95% CI 0.75-0.83) for SII, 0.73 (95% CI 0.69-0.78) for SIRI, and 0.77 (95% CI 0.73-0.81) for AISI (*P*<.01 in all cases). The cutoff values were 6.55 for CRP, 2.80 for NLR, 147.85 for PLR, 0.31 for MLR, 662.39 for SII, 1.48 for SIRI, and 443.34 for AISI. The AUC values for NLR, PLR, MLR, SII, SIRI, and AISI were statistically significantly superior to those of CRP (FDR-adjusted *P*<.05 in all cases; Table S1 in Multimedia Appendix 1), suggesting that the blood count–derived indexes were associated with psoriasis severity, although their discriminative performance remained modest. On the basis of the optimal cutoffs from the derivation cohort, the validation cohort showed consistent patterns of association (Table S2 in Multimedia Appendix 1). A consistency test was conducted in 2 groups: those with a PASI score of less than 10 and those with a PASI score of 10 or higher. The comprehensive diagnostic performance, including AUCs with 95% CIs, cutoff values, and Cohen κ analysis, is summarized in Table S3 in Multimedia Appendix 1. The results of the distribution of moderate to severe psoriasis between the SII, SIRI, PLR, and MLR and the PASI score are shown in Table S4 in Multimedia Appendix 1. There were no significant differences in the distribution of moderate to severe psoriasis between the SII, SIRI, PLR, and MLR and the PASI score (*P*>.05 in all cases). Moreover, as shown in Figure S1 in Multimedia Appendix 2, PASI scores were significantly higher among the groups with inflammatory marker values (NLR, MLR, SIRI, SII, and AISI) above the cutoffs compared to those below. However, no statistically significant difference was observed between the CRP and PLR groups. All 719 patients were divided into groups with a PASI score of less than 10 and a PASI score of 10 or higher for binary logistic regression analysis. The Hosmer-Lemeshow test showed a good fit. As shown in Table 2, the NLR, MLR, and SIRI values were higher in the group with a PASI score of 10 or higher. The odds ratios were 1.704 (95% CI 1.483-1.959), 38.132 (95% CI 12.161-119.572), and 1.914 (95% CI 1.605-2.282), respectively (*P*<.001 in all cases). The odds ratios of PLR, SII, AISI, and CRP were close to 1 (*P*<.01 in all cases). In summary, higher NLR, MLR, and SIRI values were independently associated with greater psoriasis severity independent of BMI, smoking status, and comorbidities. To account for scaling effects, log-transformed multivariable analysis was also performed, yielding robust and more interpretable odds ratios (Table S5 in Multimedia Appendix 1). These markers remained independent predictors even after further adjusting for study center, disease duration, and PsA (Table S6 in Multimedia Appendix 1). These associations remained robust after adjusting for study center in the sensitivity analysis (Table S7 in Multimedia Appendix 1). Furthermore, stratified ROC analysis showed consistent predictive performance across both hospital cohorts (Figure S2 in Multimedia Appendix 2).

**Table 2.** Binary logistic regression analysis of associations between blood count–derived inflammatory markers and psoriasis severity.

	Odds ratio (95% CI)	*P* value	Corrected odds ratio (95% CI)	*P* value
NLR^a^	1.704 (1.483-1.959)	<.001	1.683 (1.464‐1.935)	<.001
PLR^b^	1.01 (1.008-1.013)	<.001	1.011 (1.008‐1.014)	<.001
MLR^c^	38.132 (12.161-119.572)	<.001	31.815 (10.059-100.630)	<.001
SII^d^	1.002 (1.001-1.002)	<.001	1.002 (1.001-1.002)	<.001
SIRI^e^	1.914 (1.605-2.282)	<.001	1.878 (1.574-2.242)	<.001
AISI^f^	1.002 (1.002-1.003)	<.001	1.002 (1.002-1.003)	<.001
CRP^g^	1.024 (1.009-1.038)	.001	1.021 (1.007-1.036)	.004

a NLR: neutrophil-to-lymphocyte ratio.

b PLR: platelet-to-lymphocyte ratio.

c MLR: monocyte-to-lymphocyte ratio.

d SII: systemic immune-inflammation index.

e SIRI: systemic inflammation response index.

f AISI: aggregate index of systemic inflammation.

g CRP: C-reactive protein.

### Subgroup Analysis of Blood Count–Derived Inflammatory Markers

The NLR, PLR, MLR, SII, SIRI, and AISI values in the group with joint involvement were significantly higher than those in the group without joint involvement, and the MLR, SIRI, and AISI values were higher in the groups with scalp and nail involvement. The NLR, PLR, MLR, SII, SIRI, and AISI values did not show significant increases in the groups with calf and genital involvement (Table S8 in Multimedia Appendix 1). The NLR, MLR, SII, SIRI, and AISI values of patients with combined cardiovascular or metabolic diseases were significantly higher and statistically significant (Table S9 in Multimedia Appendix 1).

### Combined Analysis of Markers and Cytokines

Immunohistochemical staining of IL-1, IL-6, and IL-17 was performed on patients with psoriasis at the First Affiliated Hospital. Demographic and clinical comparison confirmed that this subcohort was representative of the full cohort (Table S10 in Multimedia Appendix 1). There were positive expressions around keratinocytes and superficial dermal inflammatory cells, as shown in Figure S3 in Multimedia Appendix 2. IL-1 and IL-17 were not expressed in the normal skin around the mole. However, by calculating the H-score, it was found that IL-17 and IL-1 had no significant correlation with the PASI score, as shown in Figures S4 and S5 in Multimedia Appendix 2.

**Figure 3.**
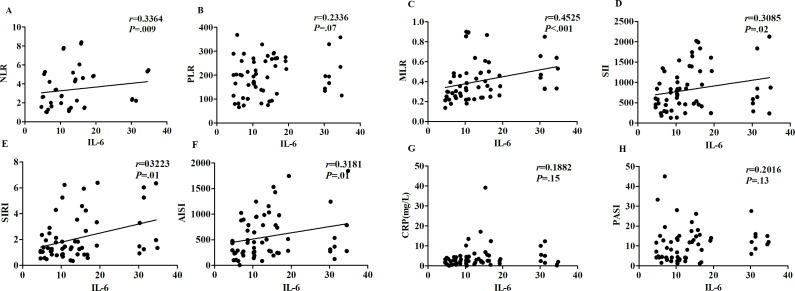
Correlation between blood count–derived indexes and interleukin (IL)-6. AISI: aggregate index of systemic inflammation; CRP: C-reactive protein; MLR: monocyte-to-lymphocyte ratio; NLR: neutrophil-to-lymphocyte ratio; PASI: Psoriasis Area and Severity Index; PLR: platelet-to-lymphocyte ratio; SII: systemic immune-inflammation index; SIRI: systemic inflammation response index.

Moreover, there was no statistically significant correlation between the blood count–derived index and IL-1 and IL-17. The correlations between NLR, MLR, SII, SIRI, and AISI and IL-6 in the skin lesions were statistically significant, with correlation coefficients of 0.3364 (*P*=.009), 0.4525 (*P*<.001), 0.3085 (*P*=.02), 0.3223 (*P*=.01), and 0.3181 (*P*=.01), respectively (Figure 3). There was no correlation between IL-6 and PASI score in the skin lesions (*P*=.13). There was no statistically significant correlation between PLR and CRP (*P*=.07 and .15, respectively).

We conducted an exploratory analysis combining IL-6, IL-1, and IL-17 with the blood count–derived indexes and found that the blood count–derived indexes combined with IL-6 in the skin lesions of patients with psoriasis had a significant correlation with the PASI score, as shown in Table 3. The correlation coefficients of the combination of IL-6 with NLR, PLR, MLR, SII, SIRI, and AISI with PASI scores were 0.66, 0.46, 0.40, 0.64, 0.56, and 0.56, respectively. The combination of IL-1 with NLR and SII and the combination of IL-17 with NLR and SII were significantly correlated with PASI scores. The correlation coefficients were 0.48, 0.50, 0.49, and 0.48, respectively. The combined multivariable model (eg, SIRI+IL-6) showed stronger associations with disease severity (AUC=0.845) and a more favorable clinical net benefit in the decision curve analysis (Figure S6 in Multimedia Appendix 2). To achieve comprehensive visual evaluation of clinical net benefit, the DCA net benefit axis was raised to 1.0 in the present analysis. Calibration analysis and a functional clinical nomogram with an individual case example further confirmed high reliability and facilitated bedside risk assessment. As summarized in the integrated correlation matrix (Figure S7 in Multimedia Appendix 2), the associations remained highly significant after FDR adjustment.

**Table 3.** The correlation between the combination of blood count–derived inflammatory markers with interleukin (IL)-6, IL-1, and IL-17 and the Psoriasis Area and Severity Index (PASI).

Interleukin and index it was combined with	Correlation with the PASI	
	*r*	*P* value
IL-6		
NLR^a^	0.66	<.001
PLR^b^	0.46	.02
MLR^c^	0.40	.05
SII^d^	0.64	.001
SIRI^e^	0.56	.003
AISI^f^	0.56	.004
IL-1		
NLR	0.48	<.001
PLR	0.38	.003
MLR	0.22	.09
SII	0.50	<.001
SIRI	0.31	.01
AISI	0.34	.007
IL-17		
NLR	0.49	<.001
PLR	0.44	.001
MLR	0.23	.08
SII	0.48	<.001
SIRI	0.31	.02
AISI	0.34	.008

a NLR: neutrophil-to-lymphocyte ratio.

b PLR: platelet-to-lymphocyte ratio.

c MLR: monocyte-to-lymphocyte ratio.

d SII: systemic immune-inflammation index.

e SIRI: systemic inflammation response index.

f AISI: aggregate index of systemic inflammation.

## Discussion

Psoriasis is driven by combined autoimmune and autoinflammatory responses, in which IL-1, IL-17, and IL-6 form an inflammatory cascade by regulating T helper 17 cells, neutrophils, and regulatory T cells [12,13]. Traditional clinical assessments such as PASI and body surface area inadequately reflect systemic inflammation, whereas cytokine-based biomarkers are costly and fail to clearly distinguish current severity from future risk [2,11]. Given the high cost and time consumption associated with specialized cytokine biomarkers, PLR, NLR, MLR, SII, SIRI, and AISI, which could be easily derived as part of a routine complete blood count, have been used in chronic inflammatory diseases, including psoriasis and psoriasis comorbidity [7,14,15]. These indicators of inflammation include neutrophil, platelet, monocyte, or lymphocyte count. Recent reports have indicated that oxidative stress, granular components, and neutrophil extracellular traps are linked to psoriasis initiation and maintenance [16]. The abnormality of the immune system in psoriasis may overactivate platelets, contributing to the disease and its cardiovascular comorbidities, with antiplatelet therapies potentially being effective [17]. A genome-wide study found that a high monocyte and neutrophil count and elevated NLR and PLR increase psoriasis risk [18], whereas patients with psoriasis vulgaris had a lower lymphocyte count than controls [3]. The excessive proinflammatory immune response in psoriasis drives the proliferation of neutrophils, platelets, and monocytes, accompanied by relative lymphocyte depletion.

Previous studies have reported elevated NLR and PLR in patients with psoriasis, although correlations with the PASI score are inconsistent [3,19]. A cohort study linked NLR to baseline PASI score and noncalcified coronary plaque burden [20]. Furthermore, SII was elevated in patients with higher PASI scores and nail or genital involvement [21]. Population-based National Health and Nutrition Examination Survey data further demonstrated positive associations between NLR, PLR, SII, and SIRI and psoriasis risk [22,23], and higher MLR and SIRI were positively associated with all-cause mortality in adults with psoriasis [23]. PLR and CRP were associated with the diagnosis of PsA, whereas SII and SIRI were not. Patients with higher PLR and SII scores were more resistant to treatment with conventional systemic agents, although no significant differences in these markers were found among psoriasis, PsA, and controls [15]. A recent meta-analysis of 36 studies confirmed significantly higher NLR, PLR, SII, and MLR in patients with psoriasis vs controls, with positive correlations between both NLR and PLR and the PASI score [24]. Inconsistent findings across studies may stem from different cohorts or populations, requiring further large-scale research. In this study, the NLR, PLR, SII, SIRI, and AISI of patients with psoriasis were higher in the group with a PASI score of 10 or higher (Figure 1), with all AUC values exceeding 0.7, supporting their potential role as exploratory severity markers (Figure 2). It was found that there was no statistically significant difference in distribution between the SII, SIRI, PLR, and MLR results and the PASI score in diagnosing moderate to severe psoriasis (Table S4 in Multimedia Appendix 1). Among them, the *P* values of the SII and AISI were .05 in both cases, which might be related to the relatively small number of patients. The higher cutoff values for the NLR, MLR, and SIRI, the higher PASI scores (Figure S1 in Multimedia Appendix 2) and the higher severity level of patients with psoriasis. The independent predictive value of these markers remained robust across different modeling approaches (Table 2). Importantly, sensitivity analysis restricted to patients without comorbidities (n=571) yielded highly consistent odds ratios, confirming that these findings were independent of systemic confounding bias (Table S11 in Multimedia Appendix 1). Comorbidity was modeled as a binary covariate; its detailed classification and modeling framework are provided in Tables S11 and S12 in Multimedia Appendix 1. On the basis of the above analysis, these findings suggest that the SIRI is associated with PASI scores and may help stratify moderate to severe psoriasis, although discriminative accuracy is not sufficiently robust for stand-alone clinical use. The robustness of these markers was further confirmed using log-transformed models, which effectively mitigated the influence of outliers and unit scaling issues. In one study, the SII was higher in patients with PsA than in those without arthritis [25]. Another study mentioned that the SII and SIRI had no significant correlation with the diagnosis of PsA, whereas SII and SIRI values were higher in patients with psoriasis involving the nails [8]. However, subgroup analyses in our study further revealed significantly higher blood count–derived indexes in patients with joint, scalp, or nail involvement, including those with cardiometabolic comorbidities (Tables S3 and S4 in Multimedia Appendix 1). These observations support the notion that blood count–derived inflammatory markers reflect systemic inflammatory burden beyond cutaneous involvement alone. The small sample size of patients with special site involvement and cardiometabolic comorbidities may introduce selection bias; future multicenter studies with larger populations are warranted to verify these findings. The PASI score is the most widely accepted and routinely used clinical index for assessing psoriasis severity. In this study, the PASI score was used as a benchmark outcome for ROC and logistic regression analyses, not as a perfect or gold-standard reflection of overall disease activity.

Currently, standardized objective biomarkers for psoriasis evaluation remain limited, and few studies have explored the combined application of blood count–derived inflammatory markers and proinflammatory cytokines. It was observed that there were positive expressions of IL-6, IL-1, and IL-17 to varying degrees around keratinocytes and superficial dermal inflammatory cells (Figure S2 in Multimedia Appendix 2). Notably, although IL-17 and IL-1 showed no significant correlation with the PASI score in the dataset, the NLR, MLR, SII, SIRI, and AISI were significantly associated with lesional IL-6 expression. This suggests that blood count–derived inflammatory markers may indirectly reflect IL-6–driven myeloid and lymphoid imbalance in psoriasis lesions. In this study, by combining IL-6, IL-1, and IL-17 with the blood count–derived inflammatory markers, it was found that there was a significant correlation with the PASI score, especially in combination with IL-6 (Table 3).

Limitations included the reliance on 2 regional centers, potential residual or unmeasured confounders, limited generalizability due to enrolling only patients requiring systemic therapy, bias from using normal perilesional skin as a control due to local microenvironmental factors, and lack of longitudinal data and external validation. Furthermore, while primary findings were validated using FDR correction, the multidimensional subgroup analyses remain exploratory and require further validation. The cytokine analysis was limited to a small subset as they may be prone to overfitting and lack sufficient generalizability; these exploratory findings should be interpreted cautiously. All these limitations should be addressed in the future.

In conclusion, blood count–derived inflammatory markers were significantly associated with the PASI score in psoriasis and correlated with lesional IL-6 expression. Moreover, the combination of blood count–derived inflammatory markers and proinflammatory factors may serve as a choice for accurately and comprehensively evaluating psoriasis severity and monitoring treatment response in the future, providing new ideas for multidimensional evaluation. As low-cost, accessible alternatives to expensive cytokine profiling, these markers support a shift from reactive to proactive, risk-stratified psoriasis management, aiding early identification of high–inflammatory burden patients. The link between blood inflammatory indexes and lesional IL-6 signaling confirms their biological relevance as surrogates of tissue-level immune dysregulation. The combined application of these markers complements traditional scores, advancing precision dermatology for more objective, individualized disease evaluation and improved long-term outcomes.

## Supplementary material

10.2196/86454 Multimedia Appendix 1 Inflammatory markers, diagnostic performance, and related analyses in patients with psoriasis.

10.2196/86454 Multimedia Appendix 2 Analyses of psoriasis inflammation, blood count-derived marker performance, cytokine expression and correlations, and multivariable predictive model evaluation.

## Abbreviations

AISI: aggregate index of systemic inflammation
AUC: area under the curve
CRP: C-reactive protein
FDR: false discovery rate
H-score: histochemistry score
IL: interleukin
MLR: monocyte-to-lymphocyte ratio
NLR: neutrophil-to-lymphocyte ratio
PASI: Psoriasis Area and Severity Index
PLR: platelet-to-lymphocyte ratio
PsA: psoriatic arthritis
ROC: receiver operating characteristic
SII: systemic immune-inflammation index
SIRI: systemic inflammation response index
