# Supplementary material for: Value of Blood Count–Derived Inflammatory Markers for Evaluating Psoriasis Severity: Pilot Cross-Sectional Observational Study
Source: Interact J Med Res. 2026 May 14;15:e86454. doi: 10.2196/86454 (PMC13175234; doi:10.2196/86454)
Supplement: Multimedia Appendix 1 [file ijmr-v15-e86454-s001.docx]

**Table S1:** Comparison of Diagnostic Performance and Cutoff Values between Derivation and Validation Cohorts.

| **Markers** | **Derivation Cohort (n=545)** |  | **Validation Cohort (n=174)** |  |
| --- | --- | --- | --- | --- |
|  | **AUC (95% CI)** | **Cutoff Value** | **AUC (95% CI)** | ***P* Value** |
| **NLR** | 0.75 (0.70–0.79) | 2.80 | 0.74 (0.65–0.83) | < .0001 |
| **PLR** | 0.70 (0.66–0.75) | 147.85 | 0.68 (0.59–0.78) | < .0001 |
| **MLR** | 0.66 (0.62–0.71) | 0.31 | 0.65 (0.55–0.75) | .0042 |
| **SII** | 0.79 (0.75–0.83) | 662.39 | 0.77 (0.68–0.85) | < .0001 |
| **SIRI** | 0.73 (0.69–0.78) | 1.48 | 0.72 (0.63–0.81) | < .0001 |
| **AISI** | 0.77 (0.73–0.81) | 443.34 | 0.76 (0.67–0.85) | < .0001 |

Cutoff values were mathematically derived from the Derivation Cohort (First Affiliated Hospital) and subsequently applied to the independent Validation Cohort (Fourth Affiliated Hospital). The comparable AUC values underscore the robustness of these inflammatory markers across different clinical settings.

Table S2: Comprehensive Diagnostic Performance and Consistency Analysis of Blood-Count-Derived Inflammatory Markers.

| **Markers** | **AUC (95% CI)** | **Cutoff Value** | **Cohen’s Kappa (95% CI)** | **Agreement Level** | **χ2 *P* value** |
| --- | --- | --- | --- | --- | --- |
| **NLR** | 0.75 (0.70–0.79) | 2.80 | 0.140 (0.000–0.285) | Slight | .0792 |
| **PLR** | 0.70 (0.66–0.75) | 147.85 | 0.102 (0.000–0.252) | Slight | .2339 |
| **MLR** | 0.66 (0.62–0.71) | 0.31 | 0.158 (0.011–0.306) | Slight | .0513 |
| **SII** | 0.79 (0.75–0.83) | 662.39 | 0.205 (0.059–0.350) | Fair | .0105 |
| **SIRI** | 0.73 (0.69–0.78) | 1.48 | 0.189 (0.043–0.334) | Slight | .0177 |
| **AISI** | 0.77 (0.73–0.81) | 443.34 | 0.170 (0.027–0.313) | Slight | .0274 |

Note: AUC and cutoff values were derived from the derivation cohort (First Affiliated Hospital, n = 545); Kappa statistics and P-values were obtained from the validation cohort (Fourth Affiliated Hospital, n = 174).

Table S3 Consistency analysis of blood-count-derived inflammatory markers and PASI in the diagnosis of moderate to severe psoriasis

| Index |  | Sensitivity(%) |  | Specificity(%) | Accuracy(%) | |  | *P* value |
| --- | --- | --- | --- | --- | --- | --- | --- | --- |
| NLR |  | 50 | | 64.5 | 56.3 |  |  | .0116 |
| PLR |  | 60.2 |  | 50 | 55.7 |  |  | .9093 |
| MLR |  | 57.1 |  | 57.9 | 57.5 |  |  | .245 |
| SII |  | 60.2 |  | 60.5 | 60.3 |  |  | .2786 |
| SIRI |  | 56.1 |  | 63.2 | 59.2 |  |  | .075 |
| AISI |  | 48 |  | 69.7 | 57.5 |  |  | .0011 |
| CRP |  | 28.6 |  | 76.3 | 49.4 |  |  | .0001 |

^PASI: Psoriasis area and severity index; CRP: C-reactive protein; NLR: neutrophil–lymphocyte ratio; MLR:monocyte-to-lymphocyte ratio; PLR:platelet–lymphocyte ratio; SII: systemic immune-inflammation index; SIRI:systemic inflammation response index; AISI:aggregate index of systemic inflammations.^

Table S4: Multivariable logistic regression analysis using log-transformed (ln) markers to address unit scaling and skewness.

| **Variable** | **Corrected Odds Ratio (OR)*** | **95% Confidence Interval (CI)** | ***P* value** |
| --- | --- | --- | --- |
| **ln(NLR)** | 4.812 | 3.251 – 7.124 | < .0001 |
| **ln(PLR)** | 6.291 | 3.986 – 9.930 | < .0001 |
| **ln(MLR)** | 3.798 | 2.518 – 5.730 | < .0001 |
| **ln(SII)** | 5.789 | 4.073 – 8.226 | < .0001 |
| **ln(SIRI)** | 4.015 | 3.012 – 5.352 | < .0001 |
| **ln(AISI)** | 3.521 | 2.707 – 4.581 | < .0001 |

Adjusted for BMI, smoking status, comorbidities, study center, disease duration, and psoriatic arthritis. The use of natural log-transformation (ln) standardizes the unit scales and mitigates the influence of skewed distributions.

**Table S5:** Extended multivariable logistic regression model demonstrating the independent predictive value of baseline inflammatory markers (using NLR as a representative model) for moderate-to-severe psoriasis (PASI ≥10), adjusted for potential clinical confounders.

| Model | Variable | Corrected Odds Ratio (OR)* | 95% Confidence Interval (CI) | *P* value |
| --- | --- | --- | --- | --- |
| Model 1 (NLR) | Disease Duration | 1.08 | 1.044 - 1.116 | < .0001 |
| Model 1 (NLR) | Psoriatic Arthritis | 1.129 | 0.599 - 2.127 | .7079 |
| Model 1 (NLR) | NLR | 1.688 | 1.460 - 1.952 | < .0001 |
| Model 2 (PLR) | Disease Duration | 1.075 | 1.041 - 1.109 | < .0001 |
| Model 2 (PLR) | Psoriatic Arthritis | 0.979 | 0.520 - 1.843 | .9486 |
| Model 2 (PLR) | PLR | 1.011 | 1.008 - 1.014 | < .0001 |
| Model 3 (MLR) | Disease Duration | 1.075 | 1.041 - 1.110 | < .0001 |
| Model 3 (MLR) | Psoriatic Arthritis | 1.243 | 0.685 - 2.256 | .4738 |
| Model 3 (MLR) | MLR | 28.208 | 8.667 - 91.811 | < .0001 |
| Model 4 (SII) | Disease Duration | 1.084 | 1.048 - 1.120 | < .0001 |
| Model 4 (SII) | Psoriatic Arthritis | 0.894 | 0.453 - 1.764 | .7461 |
| Model 4 (SII) | SII | 1.002 | 1.002 - 1.003 | < .0001 |
| Model 5 (SIRI) | Disease Duration | 1.081 | 1.046 - 1.117 | < .0001 |
| Model 5 (SIRI) | Psoriatic Arthritis | 1.092 | 0.579 - 2.057 | .7865 |
| Model 5 (SIRI) | SIRI | 1.88 | 1.566 - 2.256 | < .0001 |
| Model 6 (AISI) | Disease Duration | 1.083 | 1.048 - 1.119 | < .0001 |
| Model 6 (AISI) | Psoriatic Arthritis | 0.922 | 0.475 - 1.792 | .8113 |
| Model 6 (AISI) | AISI | 1.002 | 1.002 - 1.003 | < .0001 |

Table S6: Sensitivity analysis using binary logistic regression adjusting for study center to predict disease severity of psoriasis (PASI ≥10).

| **Variables** | **Corrected Odds Ratio (OR)*** | **95% Confidence Interval (CI)** | ***P* value** |
| --- | --- | --- | --- |
| **NLR** | 1.671 | 1.452 - 1.923 | < .0001 |
| **PLR** | 1.011 | 1.008 - 1.014 | < .0001 |
| **MLR** | 29.030 | 9.105 - 92.561 | < .0001 |
| **SII** | 1.002 | 1.002 - 1.002 | < .0001 |
| **SIRI** | 1.857 | 1.556 - 2.217 | < .0001 |
| **AISI** | 1.002 | 1.002 - 1.003 | < .0001 |
| **CRP** | 1.020 | 1.005 - 1.035 | .0071 |

Table S7 Clinical parameters were compared based on special parts

| Index |  | Joint |  |  |  | Nail |  |  |
| --- | --- | --- | --- | --- | --- | --- | --- | --- |
|  |  | no (660) | yes (n=59) | *P* value |  | no (548) | yes (171) | *P* value |
| PASI score, Median (P25, P75) | | 9.05 (5.40, 16.20) | 14.40 (8.50, 28.00) | < .001 |  | 9.00(5.20, 16.1) | 10.30 (6.00, 18.40) | .006 |
| NLR, Median (P25, P75) | | 2.30 (1.72, 3.05) | 2.58 (1.91, 5.18) | .002 |  | 2.32 (1.72, 3.17) | 2.35 ( 1.80, 3.11) | .584 |
| PLR, Median (P25, P75) | | 140.40 (111.50, 179.00) | 170.20( 135.00, 258.10) | < .001 |  | 145.20 (112.90, 183.90) | 138.50 (115.50, 176.50) | .683 |
| MLR, Median (P25, P75) | | 0.28 ( 0.22, 0.37) | 0.35 (0.26, 0.51) | .001 |  | 0.29 (0.22, 0.37) | 0.30 ( 0.24, 0.43) | .048 |
| SII, Median (P25, P75) | | 618.20 (434.20, 898.40) | 915.90 (568.40, 1635.00) | < .001 |  | 624.60 (433.90, 916.60) | 641.40 (459.00, 965.50) | .367 |
| SIRI, Median (P25, P75) | | 1.27 (0.88, 1.85) | 1.87 (1.01, 3.14) | .001 |  | 1.27 (0.87, 1.89) | 1.38 (0.98, 2.19) | .051 |
| AISI, Median (P25, P75) | | 344.10 (225.40, 532.80) | 645.20 (299.60, 1112.00) | < .001 |  | 347.7 (221.9, 543.2) | 382.00 ( 248.1, 673.50) | .042 |
| Index |  | Scalp |  |  |  | Calf |  |  |
|  |  | no (n=293) | yes (n=426) | *P* value |  | no(n=613) | yes (n=106) | *P* value |
| PASI score,Median (P25, P75) | | 9.00 (5.40, 15.00) | 9.60 (5.60, 18.00) | .087 |  | 9.30 (5.40, 16.50) | 9.60 (6.00, 18.00) | .303 |
| NLR, Median (P25, P75) | | 2.26 (1.70, 3.06) | 2.38 (1.77, 3.22) | .115 |  | 2.31 (1.73, 3.13) | 2.40 ( 1.77, 3.20) | .426 |
| PLR, Median (P25, P75) | | 143.10 (108.60, 185.40) | 143.80 ( 114.40, 179.30) | .544 |  | 143.80 (112.90, 181.40) | 143.40 (118.40, 185.20) | .515 |
| MLR, Median (P25, P75) | | 0.28 ( 0.21, 0.35) | 0.30 (0.23, 0.40) | .024 |  | 0.28 (0.22, 0.38) | 0.32 ( 0.25, 0.43) | .054 |
| SII, Median (P25, P75) | | 609.30 (422.60, 886.20) | 647.00 (450.30,945.60) | .168 |  | 628.10 (438.20, 916.40) | 614.20 (454.60, 957.00) | .72 |
| SIRI, Median (P25, P75) | | 1.23 (0.86, 1.81) | 1.36 (0.92, 2.10) | .043 |  | 1.27 (0.88, 1.95) | 1.38 (0.95, 2.08) | .339 |
| AISI, Median (P25, P75) | | 321.40 (214.00, 534.60) | 375.70 (234.20, 594.10) | .051 |  | 349.40 (228.30, 558.50) | 375.40 ( 228.50, 603.50) | .53 |
| Index |  | genital |  |  |  |  |  |  |
|  |  | no (n=636) | yes (n=83) | *P* value |  |  |  |  |
| PASI score,Median (P25, P75) | | 9.00 (5.40, 16.08) | 15.90 (6.00, 27.90) | < .001 |  |  |  |  |
| NLR, Median (P25, P75) | | 2.30 (1.73, 3.09) | 2.36 (1.80, 3.56) | .084 |  |  |  |  |
| PLR, Median (P25, P75) | | 143.00 (114.20, 179.50) | 146.90 ( 108.60, 193.60) | .373 |  |  |  |  |
| MLR, Median (P25, P75) | | 0.29 ( 0.22, 0.37) | 0.31 (0.25, 0.43) | .077 |  |  |  |  |
| SII, Median (P25, P75) | | 618.20 (436.20, 918.40) | 673.50 (497.30,944.50) | .22 |  |  |  |  |
| SIRI, Median (P25, P75) | | 1.27 (0.89, 1.92) | 1.41 (0.95, 2.22) | .23 |  |  |  |  |
| AISI, Median (P25, P75) | | 353.30 (223.40, 566.00) | 361.30 (261.80, 611.20) | .367 |  |  |  |  |

PASI: Psoriasis area and severity index; NLR: neutrophil–lymphocyte ratio; MLR:monocyte-to-lymphocyte ratio; PLR:platelet–lymphocyte ratio;

SII: systemic immune-inflammation index; SIRI:systemic inflammation response index; AISI:aggregate index of systemic inflammations.

Table S8 Comparison of clinical parameters in the presence or absence of cardiovascular or metabolic diseases

| Index | cardiovascular or metabolic diseases | | |
| --- | --- | --- | --- |
|  | no (622) | yes (n=97) | *P* value |
| PASI score,Median (P25, P75) | 8.95 (5.18, 15.93) | 14.40 (9.40, 24.80) | < .001 |
| NLR, Median (P25, P75) | 2.28 (1.72, 3.04) | 2.87 (1.87, 4.18) | < .001 |
| PLR, Median (P25, P75) | 140.60 (112.60, 181.60) | 148.40( 122.00, 185.00) | .094 |
| MLR, Median (P25, P75) | 0.28 ( 0.22, 0.36) | 0.35 (0.26, 0.49) | < .001 |
| SII, Median (P25, P75) | 614.40 (433.30, 899.60) | 717.80 (525.60, 1148.00) | .003 |
| SIRI, Median (P25, P75) | 1.27 (0.85, 1.82) | 1.62 (1.05, 2.91) | < .001 |
| AISI,Median (P25, P75) | 344.30 (222.30, 537.60) | 451.10 (279.40, 798.20) | < .001 |

Table S9: Comparison of baseline characteristics between the IHC sub-cohort (n=60) and the total study cohort (n=719).

| **Characteristics** | **IHC Sub-cohort (n=60)** | **Total Cohort (n=719)** | ***P* value** |
| --- | --- | --- | --- |
| **Age** (years) | 43.00 ± 15.6 | 44.71 ± 15.5 | .993 |
| **Gender** (male, %) | 41 (68.3%) | 524 (72.9%) | .448 |
| **BMI** (kg/m^2^) | 22.14 ± 4.2 | 22.50 ± 4.4 | .542 |
| **PASI score** | 13.12 (9.8, 18.5) | 9.80 (5.4, 17.5) | .126 |

No significant differences were observed, indicating that the IHC sub-cohort is representative of the broader psoriasis population in this study.

Table S10: Sensitivity analysis of inflammatory markers in patients without any comorbidities (n=571).

| **Markers** | **Full Model (Adjusted for Comorbidity)OR (95% CI)** | **No-Comorbidity Subgroup (n=571)OR (95% CI)** | **Consistency** |
| --- | --- | --- | --- |
| **NLR** | 1.670 (1.452–1.922) | 1.625 (1.394–1.895) | **High** |
| **PLR** | 1.011 (1.008–1.014) | 1.012 (1.008–1.015) | **High** |
| **MLR** | 28.551 (8.955–91.026) | 28.305 (7.787–102.879) | **High** |
| **SII** | 1.002 (1.002–1.002) | 1.002 (1.001–1.002) | **High** |
| **SIRI** | 1.854 (1.553–2.212) | 1.775 (1.466–2.149) | **High** |
| **AISI** | 1.002 (1.002–1.003) | 1.002 (1.002–1.003) | **High** |

*P* < .0001for all reported Odds Ratios.

Table S11: Detailed composition of comorbidities in the study population (n=719).

| **Category** | **Specific Condition** | **Frequency (n)** | **Percentage (%)** |
| --- | --- | --- | --- |
| **Metabolic/CVMD** | Hypertension | 68 | 9.46% |
|  | Type 2 Diabetes Mellitus | 42 | 5.84% |
|  | Dyslipidemia | 51 | 7.09% |
|  | Hyperuricemia | 33 | 4.59% |
|  | Fatty Liver Disease | 24 | 3.34% |
| **Others** | Chronic Gastritis | 12 | 1.67% |
|  | Others (Benign thyroid nodules, etc.) | 9 | 1.25% |
| **Total Unique Patients** | **With at least one comorbidity** | **148** | **20.58%** |

In the multivariable logistic regression models, comorbidity was treated as a binary categorical variable (0 = No comorbidities; 1 = Presence of one or more comorbidities).

Table S12: Detailed classification and modeling of reported comorbidities.

| **Category** | **Defined Conditions** | **Modeling Method** |
| --- | --- | --- |
| **Metabolic/CVMD** | Hypertension, Type 2 Diabetes, Dyslipidemia, Hyperuricemia, Fatty Liver | Binary (1 = Presence of \geq 1 condition; 0 = None) |
| **Exclusionary Criteria** | Acute infection, hematologic malignancies, significant anemia, bone marrow suppression | **Excluded** (Patients with these conditions were not enrolled) |

Table S13: Summary of Multiple Comparison Adjustments using False Discovery Rate (FDR) for Primary and Exploratory Analyses.

| Marker | Target | r | *P* | q |
| --- | --- | --- | --- | --- |
| NLR | PASI (Corr) | 0.416285 | ＜.001 | ＜0.001 |
| PLR | PASI (Corr) | 0.350272 | ＜.001 | ＜0.001 |
| MLR | PASI (Corr) | 0.334734 | ＜.001 | ＜0.001 |
| SII | PASI (Corr) | 0.466535 | ＜.001 | ＜0.001 |
| SIRI | PASI (Corr) | 0.411565 | ＜.001 | ＜0.001 |
| AISI | PASI (Corr) | 0.446508 | ＜.001 | ＜0.001 |
| NLR | IL-6 (Corr) | 0.347429 | .006 | 0.007 |
| PLR | IL-6 (Corr) | 0.281502 | .028 | 0.028 |
| MLR | IL-6 (Corr) | 0.473045 | ＜.001 | ＜0.001 |
| SII | IL-6 (Corr) | 0.408981 | .001 | 0.001 |
| SIRI | IL-6 (Corr) | 0.479072 | ＜.001 | ＜0.001 |
| AISI | IL-6 (Corr) | 0.482457 | ＜.001 | ＜0.001 |

The Benjamini-Hochberg procedure was used to control the False Discovery Rate (FDR) and mitigate Type I errors. Raw *P*-values and adjusted q-values are reported. Primary associations (NLR, SIRI, SII, and AISI vs. PASI) remained robustly significant after FDR control (q < 0.001). Secondary subgroup and cytokine analyses are exploratory and intended for hypothesis generation.
