# Supplementary material for: Value of Blood Count–Derived Inflammatory Markers for Evaluating Psoriasis Severity: Pilot Cross-Sectional Observational Study
Source: Interact J Med Res. 2026 May 14;15:e86454. doi: 10.2196/86454 (PMC13175234; doi:10.2196/86454)
Supplement: Multimedia Appendix 2 [file ijmr-v15-e86454-s002.docx]

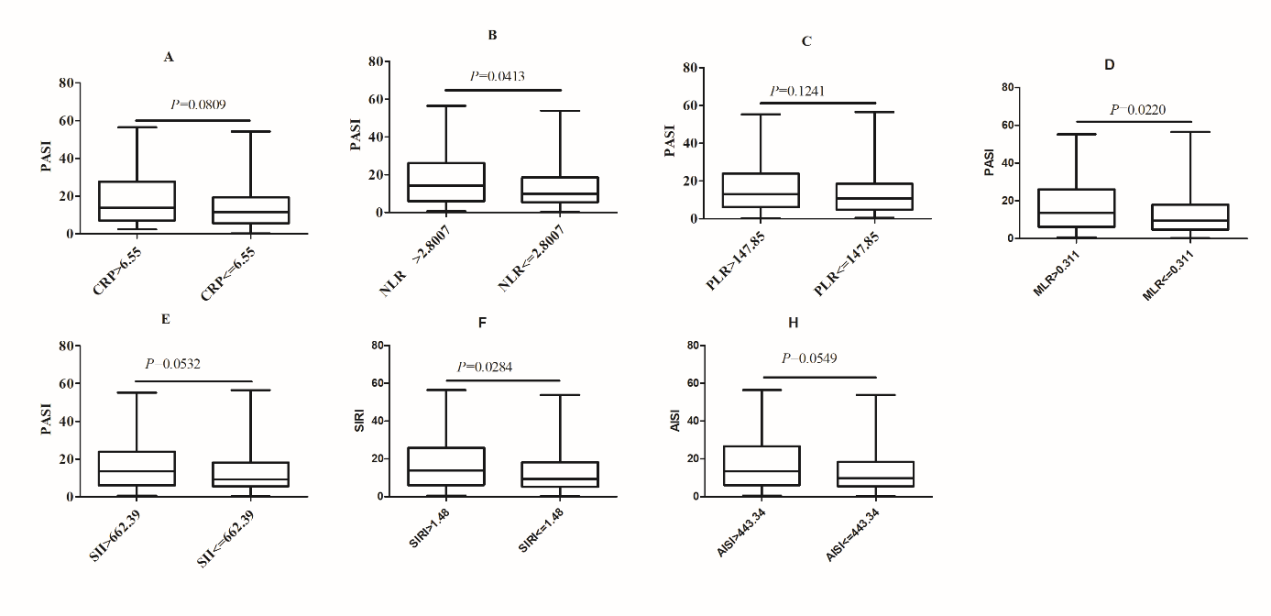


Figure S1 the differential expression of PASI value in different psoriasis inflammation degree

PASI: Psoriasis area and severity index; CRP: C-reactive protein; NLR: neutrophil–lymphocyte ratio; MLR:monocyte-to-lymphocyte ratio; PLR:platelet–lymphocyte ratio; SII: systemic immune-inflammation index; SIRI:systemic inflammation response index; AISI:aggregate index of systemic inflammations.


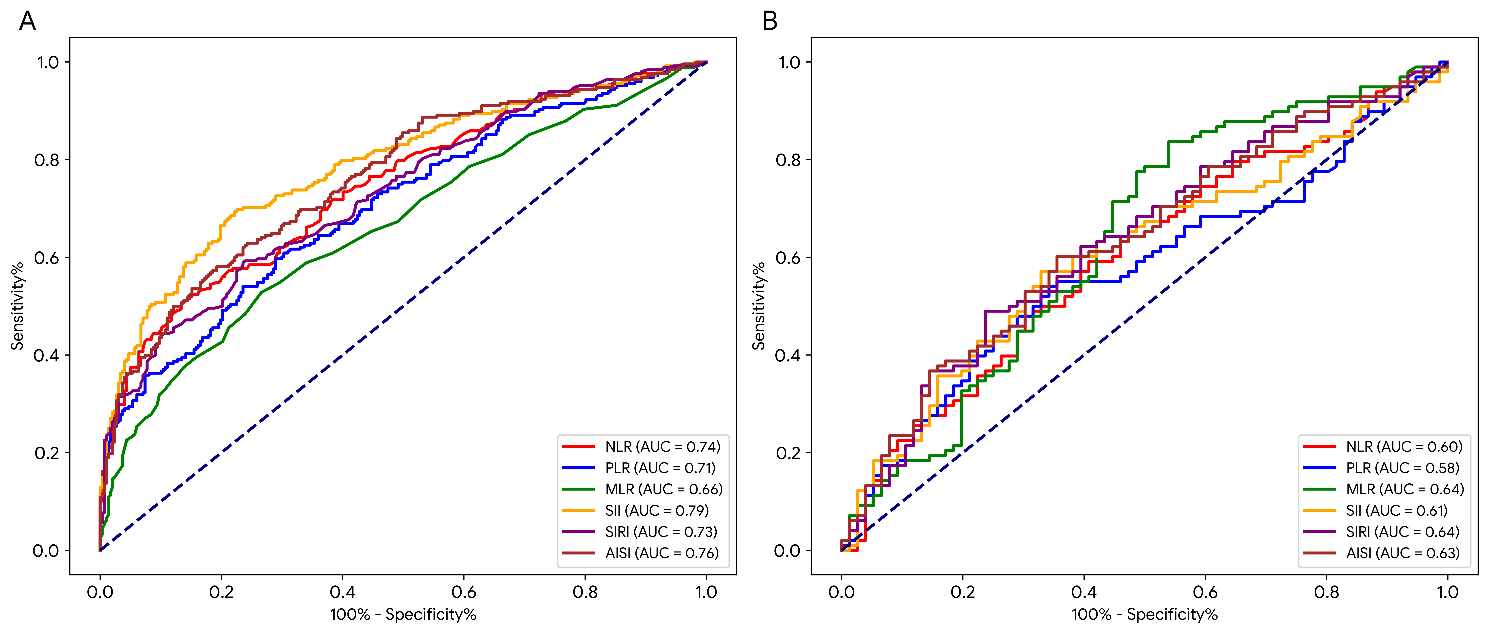


Figure S2. Stratified Receiver Operating Characteristic (ROC) curve analysis of blood-count-derived inflammatory markers for predicting moderate-to-severe psoriasis (PASI ≥10).

(A) ROC curves and corresponding Area Under the Curve (AUC) values for the patient cohort from the First Affiliated Hospital. (B) ROC curves and AUC values for the patient cohort from the Fourth Affiliated Hospital. The consistent predictive performance across both independent centers demonstrates that the discriminative ability of these markers is robust against institutional heterogeneity. NLR: neutrophil-to-lymphocyte ratio; PLR: platelet-to-lymphocyte ratio; MLR: monocyte-to-lymphocyte ratio; SII: systemic immune-inflammation index; SIRI: systemic inflammation response index; AISI: aggregate index of systemic inflammations.


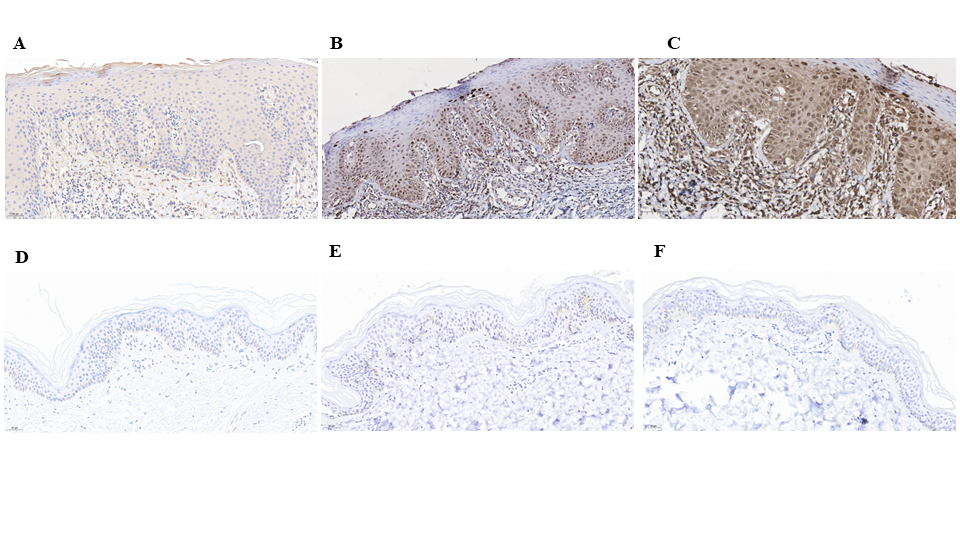


Figure S3 Immunohistochemical expression of IL-17, IL-1 and IL-6

A: Expression of IL-6 in psoriasis lesions; B: Expression of IL-17 in psoriasis lesions; C: Expression of IL-1 in psoriasis lesions; D: Expression of IL-6 in the normal skin around the mole; E: Expression of IL-17 in the normal skin around the mole; F: Expression of IL-1 in the normal skin around the mole. Scale :50 μ m.


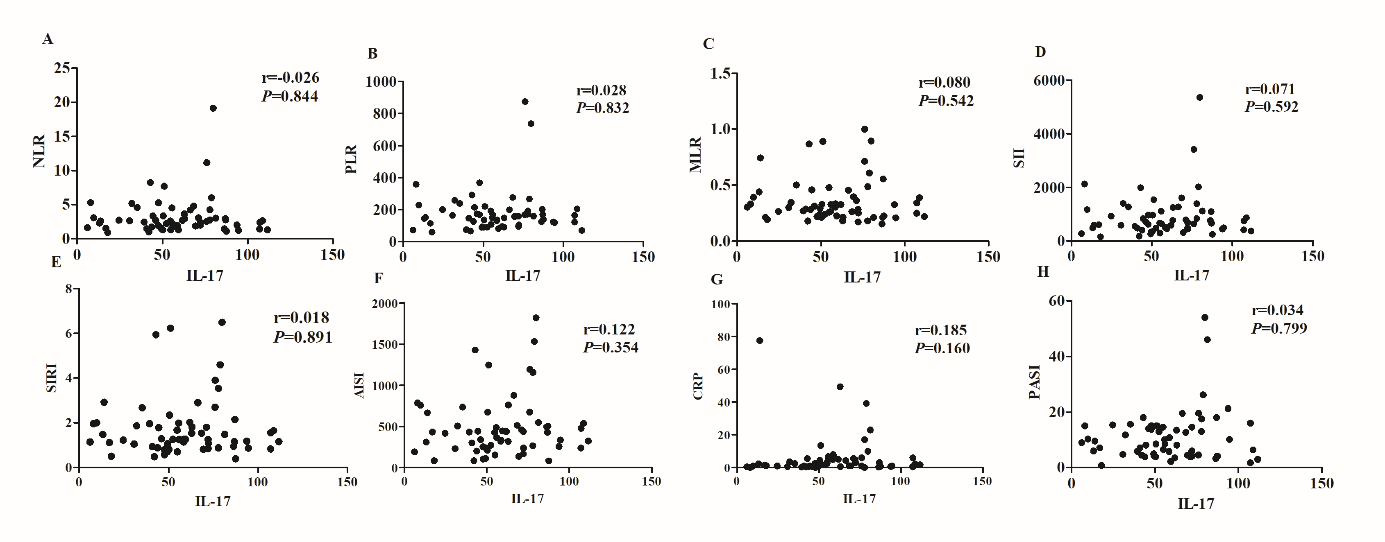


Figure S4 Correlation of blood cell ratio derived index and IL-17

NLR: neutrophil–lymphocyte ratio; MLR:monocyte-to-lymphocyte ratio; PLR:platelet–lymphocyte ratio; SII: systemic immune-inflammation index; SIRI:systemic inflammation response index; AISI:aggregate index of systemic inflammations.


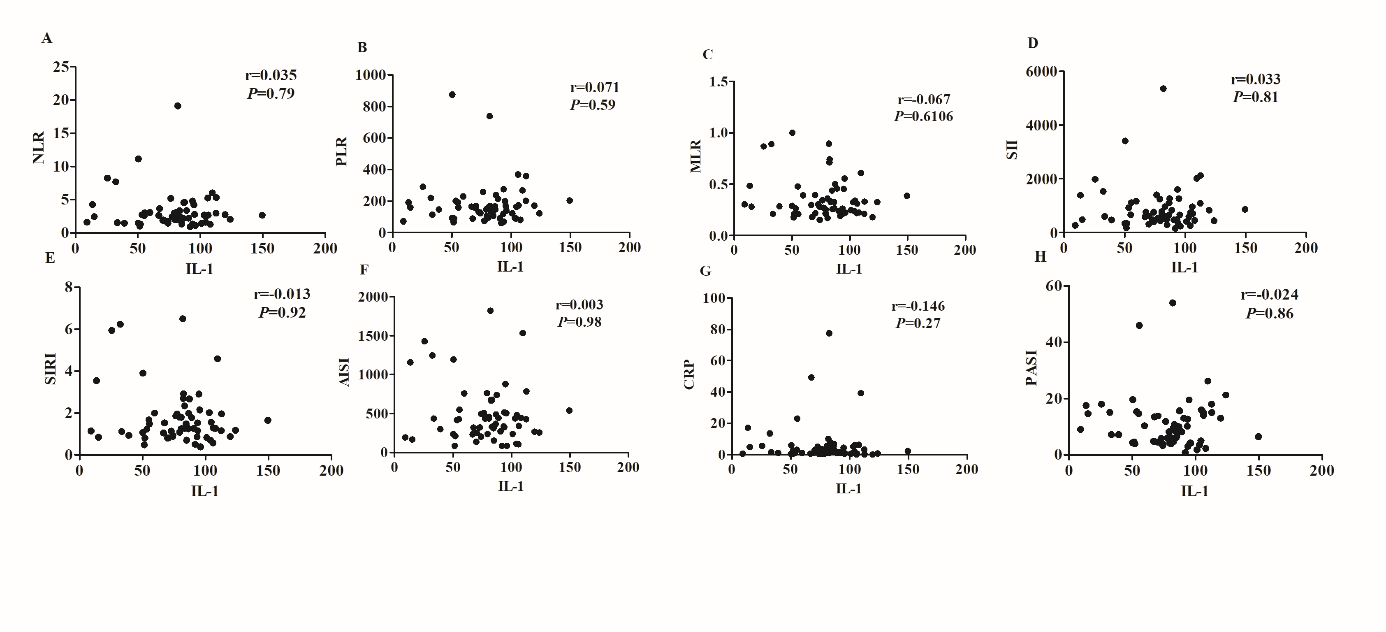
、

Figure S5 Correlation of blood cell ratio derived index and IL-1

NLR: neutrophil–lymphocyte ratio; MLR:monocyte-to-lymphocyte ratio; PLR:platelet–lymphocyte ratio; SII: systemic immune-inflammation index; SIRI:systemic inflammation response index; AISI:aggregate index of systemic inflammations.


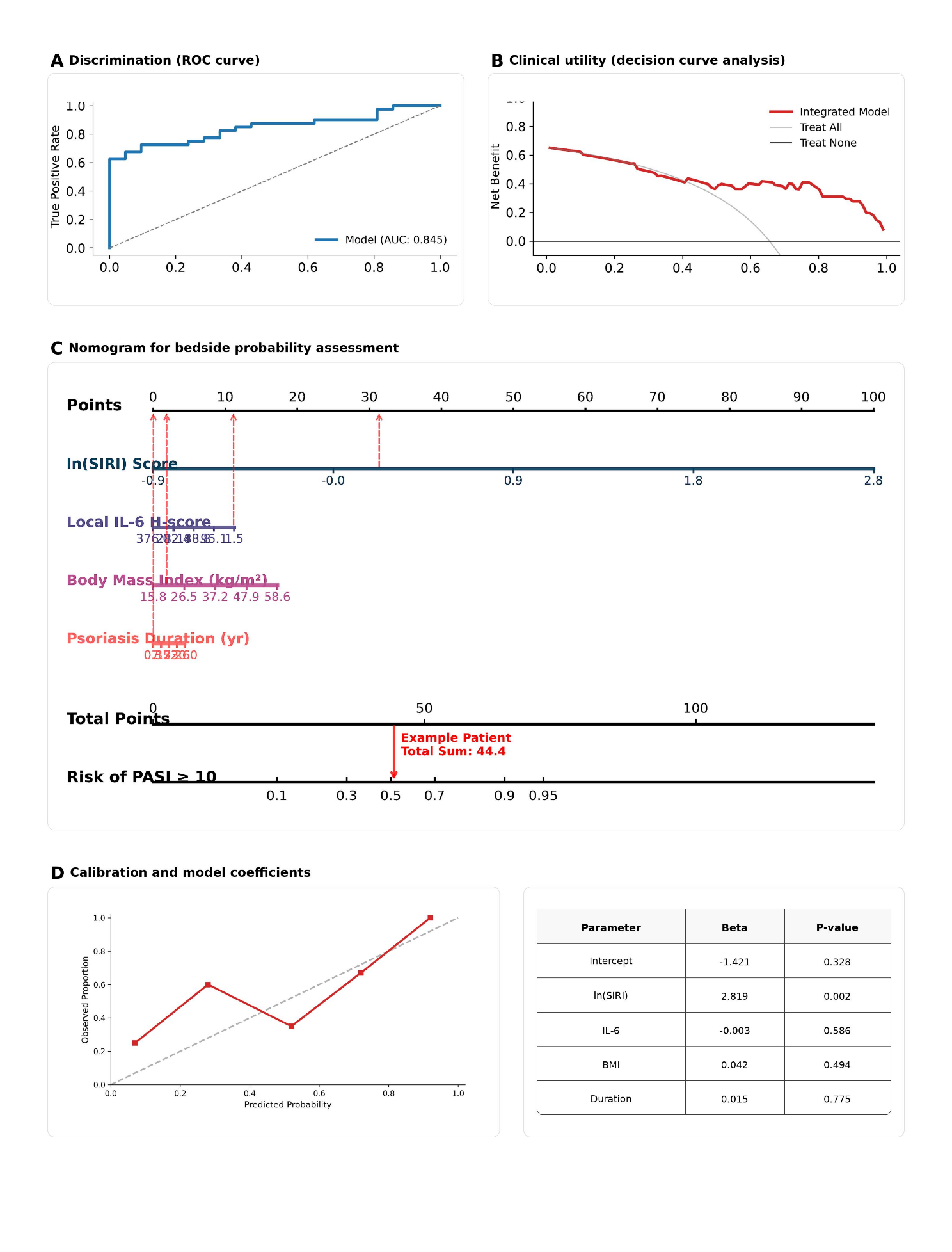


Figure S6. Evaluation of the Integrated Multivariable Predictive Model for Psoriasis Severity.

**(A) Discrimination (ROC Curves):** Comparison between the baseline model and the integrated model (ln(SIRI) + IL-6 + covariates) in the IHC sub-cohort (n=61). The combined model achieved an **AUC of 0.845 (95% CI: 0.741–0.933)**. 95% CIs were determined by 1,000 bootstrap resamples. **(B) Clinical Utility (DCA):** Decision curve analysis demonstrating the clinical net benefit of the integrated model. The Y-axis (Net Benefit) is **raised to 1.0** for comprehensive visualization. **(C) Clinical Nomogram:** Individualized risk assessment tool for PASI \geq 10. The red dashed lines illustrate a representative case example to facilitate clinical interpretation. **(D) Reliability (Calibration):** Calibration plot showing the agreement between predicted and observed risks. The Hosmer-Lemeshow test (*P* >.05) indicates high model reliability. **(E) Regression Parameters:** Multivariable logistic regression coefficients (Beta) and significance levels (*P*-values) for all predictors in the integrated model.

*Note: ln(SIRI), natural log-transformed systemic inflammation response index; IL-6, interleukin-6; AUC, area under the curve; CI, confidence interval; DCA, decision curve analysis.*


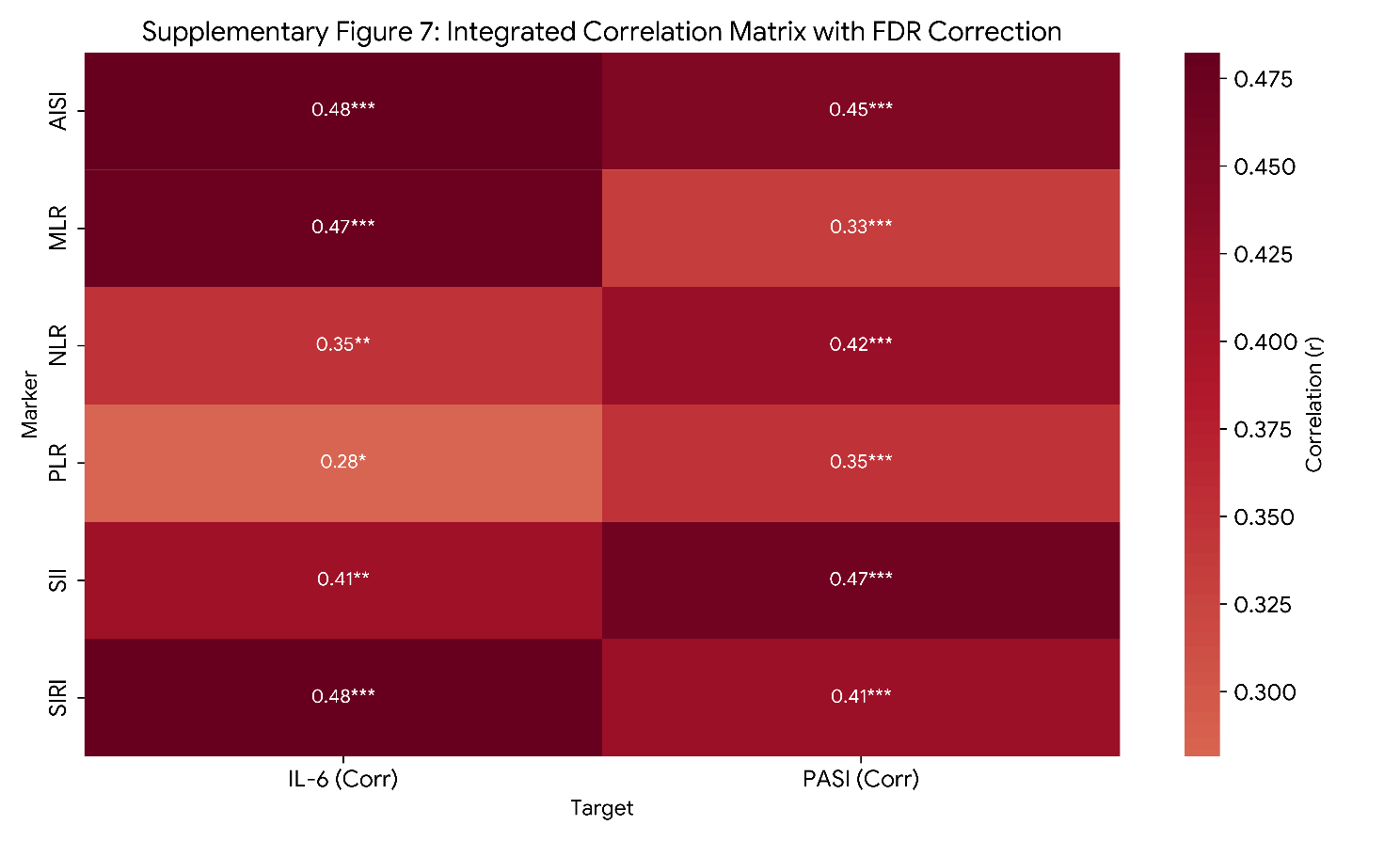


Figure S7: Integrated Correlation Matrix of Inflammatory Markers with Disease Severity and Pro-inflammatory Factors. This heatmap illustrates the Spearman correlation coefficients (r) between blood-count-derived inflammatory markers and the Psoriasis Area and Severity Index (PASI) in the total cohort (n=719), as well as IL-6 levels in the skin lesions of the sub-cohort (n=60). Significance levels are annotated with asterisks based on False Discovery Rate (FDR) adjusted p-values (q-values) using the Benjamini-Hochberg procedure: * q < 0.05, ** q < 0.01, *** q < 0.001. All primary associations remained highly significant after controlling for multiple comparisons, underscoring the robustness of the findings.
